# Discontinuation rates of intrauterine contraception due to unfavourable bleeding: a systematic review

**DOI:** 10.1186/s12905-022-01657-6

**Published:** 2022-03-21

**Authors:** Dustin Costescu, Rajinder Chawla, Rowena Hughes, Stephanie Teal, Martin Merz

**Affiliations:** 1 Department of Obstetrics and Gynaecology, McMaster University, Hamilton, ON Canada; 2 AccuScript Consultancy, Ludhiana, Punjab India; 3 AccuScript Consultancy, Reading, UK; 4 Department of Obstetrics and Gynecology, University Hospitals Cleveland Medical Center, Cleveland, OH USA; 5 Medical Affairs and Pharmacovigilance, Pharmaceuticals MA TA Women’s Health Care, Bayer AG, Building S101, 10/244, 13342 Berlin, Germany

**Keywords:** Contraception, Discontinuation, Intrauterine device, Menstrual bleeding, Satisfaction

## Abstract

**Objective:**

Levonorgestrel-releasing intrauterine devices (LNG-IUDs) and copper intrauterine devices (Cu-IUDs) offer long-acting contraception; however, some women may discontinue use within the first year due to bleeding pattern changes, limiting their potential. This systematic literature review investigated whether differences in bleeding profiles influence continuation rates in women in America, Europe and Australia.

**Methods:**

Searches performed in PubMed and Embase were screened to identify publications describing bleeding patterns and rates of early IUC removal/discontinuation or continuation, descriptions of bleeding patterns, reasons for discontinuation, and patient satisfaction, acceptability and tolerability for LNG-IUDs and Cu-IUDs published between January 2010 and December 2019. The results were further restricted to capture citations related to ‘Humans’ and ‘Females’. The review was limited to studies published from 2010 onwards, as changing attitudes over time mean that results of studies performed before this date may not be generalizable to current practice.

**Results:**

Forty-eight publications describing 41 studies performed principally in the USA (n = 17) and Europe (n = 13) were identified. Publications describing bleeding patterns in LNG-IUD users (n = 11) consistently observed a reduction in bleeding in most women, whereas two of three studies in Cu-IUD users reported heavy bleeding in approximately 40% of patients. Rates of discontinuation for both devices ranged widely and may be as high as 50% but were lower for LNG-IUDs versus Cu-IUDs. Discontinuation rates due to bleeding were consistently higher for Cu-IUDs versus LNG-IUDs.

**Conclusions:**

Bleeding is a common reason for discontinuation of Cu-IUDs and LNG-IUDs. The more favourable bleeding pattern observed in LNG-IUD users may be associated with a lower rate of early discontinuation of LNG-IUDs versus Cu-IUDs.

**Supplementary Information:**

The online version contains supplementary material available at 10.1186/s12905-022-01657-6.

## Background

Intrauterine devices (IUDs) or intrauterine contraceptives (IUCs) are highly efficacious, highly acceptable and cost-effective [1]. Women relying on IUDs have substantially lower rates of unintended pregnancy than those using short-acting and non-hormonal user-dependent methods of contraception [2]. IUD use decreases unintended births, abortion, adolescent pregnancy and health care expenditure [3].

There are two main types of IUDs: levonorgestrel-releasing intrauterine devices (LNG-IUDs) and copper IUDs (Cu-IUDs). Although both are highly effective, they differ in key characteristics and mechanism of action [4]. Both IUDs are associated with medically benign changes to menstrual bleeding pattern, and it is widely accepted that LNG-IUDs tend to reduce menstrual flow and dysmenorrhoea, whereas in Cu-IUD users, increased menstrual flow and dysmenorrhoea have been reported [5, 6].

Although IUCs have higher continuation and satisfaction rates than other contraceptive methods, a proportion of users who do not desire pregnancy discontinue use within the first year (early discontinuation), generally due to side-effects such as cramping and bleeding [7]. In addition, the experience and satisfaction of women play an important role in whether they request early IUC removal. Early discontinuation typically results in uptake of less-effective contraception such as traditional methods (e.g. periodic abstinence or withdrawal) [8, 9]. It is therefore important to better understand the incidence of and contributors to early discontinuation. Given the recognised difference in bleeding profile between LNG-IUDs and Cu-IUDs, this systematic literature review was undertaken to investigate whether bleeding profiles influence continuation rates and the extent to which women request removal of either type of device as a result of unfavourable changes in menstrual bleeding. In order to identify all relevant evidence relating to this clinical issue, the review aimed to include a wide variety of different study types and not be limited by design, subject characteristics or definitions for study endpoints. With this in mind, performance of a meta-analysis was not planned.

## Methods

The systematic review is reported in accordance with the Preferred Reporting Items for Systematic Reviews and Meta-Analysis Protocol (PRISMA-P) guidelines [10]. Searches were performed in PubMed, and Embase to identify all relevant English language publications from 1 January 2000 to 28 November 2019. The search strategy aimed to identify publications describing bleeding patterns and discontinuation rates in women using LNG-IUDs or Cu-IUDs (see Additional file 1: Appendix 1). Publications were screened to identify studies (of any design) in healthy adult women reporting rates of early IUC removal/discontinuation or continuation, descriptions of bleeding patterns, reasons for discontinuation, and patient satisfaction, acceptability and tolerability. Specifically, the reviewers sought to identify prevalence of favourable and unfavourable bleeding patterns, differences in bleeding patterns among devices, variables that correlate with bleeding, and the association between bleeding and discontinuation. Publications reporting outcomes for women using IUCs for therapeutic indications and studies only describing contraceptive benefits were excluded (see Additional file 1: Appendix 2 for inclusion and exclusion criteria).

Screening based on title and abstract was performed by one researcher (Gaganpreet Kaur of Accuscript Consultancy) and all excluded references were checked by a second researcher (RC). Full papers were obtained and were screened by the researcher. A senior researcher (RH) reviewed the results for authentication and resolution of any uncertainties. Data from included references were extracted by one researcher (Gaganpreet Kaur) and were reviewed by a second researcher (RC). It was considered that continuation/discontinuation rates due to bleeding may be influenced by cultural differences in the perceptions regarding bleeding, with bleeding being seen as favourable and amenorrhoea being viewed negatively in some cultures, including countries in Asia, the Middle East and Africa. It was therefore decided at full-text review to exclude publications from Asia, the Middle East and Africa. Similarly, the review was limited to studies published from 2010 onwards, as changing attitudes over time mean that results of studies performed before this date may not be generalisable to current practice.

## Results

### Overview of selected studies

**Fig. 1:**
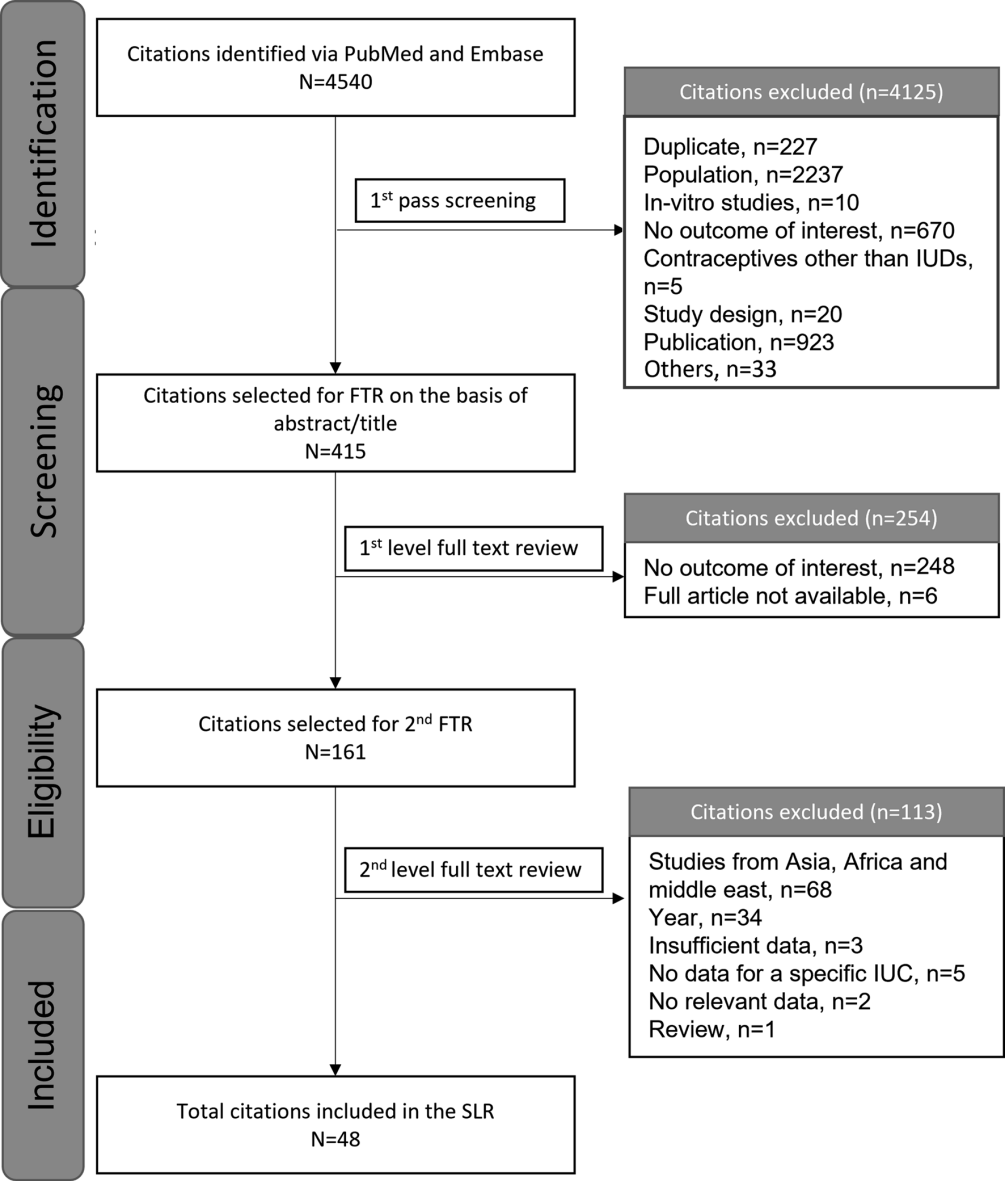
PRISMA for the studies included in the systematic literature review. FTR, full-text review; IUC, intrauterine contraceptive; IUD, intrauterine device; PRISMA, Preferred Reporting Items for Systematic Reviews and Meta-Analyses; SLR, systematic literature review

A total of 53 publications met the inclusion criteria (Fig. 1); however, five publications did not report data according to IUC type and are therefore not discussed further; the remaining 48 publications are summarised in Table 1. Most publications were for distinct studies, but the single-arm phase III trial of LNG-IUS, ACCESS, was reported in four publications [11–14]; a single-arm European study of LNG-IUD was reported in two publications [15, 16]; and results from a randomised clinical trial (RCT) comparing an LNG-IUD 13.5 mg with 19.5 mg was described in two publications [17, 18]. Two publications by Korjamo et al. report data from an overlapping cohort of women using an LNG-IUD post medical termination of pregnancy [19, 20]. In addition, four publications reported results from the prospective, comparative cohort study, Contraceptive CHOICE Project; these each report data for different (but likely overlapping) cohorts so are considered as separate studies[21–24]. Of the individual studies, 17 were performed in the USA, 13 in individual European countries, and 5 were multinational; 4 were performed in South American countries, 2 in Australia and 1 in Canada. Most studies (70%) included both nulliparous and parous women. As anticipated, individual studies were very heterogeneous in their design, patient populations, descriptions of bleeding patterns, definitions for discontinuation, and measures of treatment satisfaction. It was therefore not considered relevant to assess the feasibility of performing a meta-analysis. A risk of bias assessment rated 8 of 17 case control/RCTs and 10 of 29 cohort studies as being of good quality (Additional file 1: Appendix 3).

**Table 1:** Summary of publications included in the review

References	Country	Study design	Patients, N	Intervention(s)
*LNG-IUD*				
Shimoni et al. [25]	USA	Prospective comparative observational study	131	LNG-IUD, 13.5 mg (Skyla) early vs late menstrual cycle insertion
Teal et al. [14]	USA	Single-arm phase III study, ACCESS IUS	1751^a^	LNG-IUD, 52 mg (Liletta)
Darney et al. [11]	USA	Secondary analysis of phase III study, ACCESS IUS	1751^a^	LNG-IUD, 52 mg (Liletta)
Schreiber et al. [13]	USA	Secondary analysis of phase III study, ACCESS IUS	1751^a^	LNG-IUD, 52 mg (Liletta)
Eisenberg et al. [12]	USA	Single-arm phase III study, ACCESS IUS	1751^a^	LNG20-IUD, 52 mg (Liletta)
Neri et al. [26]	Italy	Prospective single-arm	25	LNG-IUD, 6 µg/day (Jaydess)
Vaitsiakhovich et al. [27]	Germany	Analysis of data from an observational study and RCT	1860, 1607	LNG-IUD, 52 mg (Mirena)
Carvalho et al. [28]	Brazil	Prospective, observational, single-arm	231	LNG-IUD, 20 µg/day (Mirena)
Korjamo et al. [19] ^b^	Finland	RCT	159	LNG-IUD (Mirena) immediate vs late insertion following MTOP
Korjamo et al. [20] ^b^	Finland	RCT (same study as Korjamo et al. [20])	267	LNG-IUD (Mirena) immediate vs late insertion following MTOP
Cristobal et al. [29]	Spain	Prospective, observational, single-arm	201	LNG-IUD, 52 mg
Whitaker et al. [30]	USA	RCT	42	LNG-IUD, immediate vs late insertion following caesarean delivery
Stoegerer-Hecher et al. [32]	Austria	Cross-sectional	415	LNG-IUD (Mirena)
Gemzell-Danielsson et al. [15]^c^	Finland, France, Ireland and Sweden	Prospective single-arm	204	LNG-IUD
Heikinheimo et al. [16]^c^	Finland, France, Ireland and Sweden	Prospective, single-arm (same study as Gemzell-Danielsson et al. [15])	204	LNG-IUD
Armitage et al. [31]	UK	Prospective, observational	100	LNG-IUD
Nelson et al. [18]^d^	Multinational	RCT	1432 vs 1452	LNG-IUD 13.5 mg vs 19.5 mg
Gemzell-Danielsson et al. [17] ^d^	Multinational	Post-hoc analysis of phase III RCT (Nelson et al. [18])	1432 vs 1452	LNG-IUD 13.5 mg vs 19.5 mg
*Cu-IUD*				
Yaron et al. [33]	Switzerland	Retrospective, observational	207	Cu-IUD, Ballerine MIDI
Sanders et al. [34]	USA	Prospective, longitudinal, observational	77	Cu-IUD, CuT380A
Bateson et al. [35]	Australia	Prospective, observational	211	Cu-IUD (TT380 short or long, or a multiload device)
Jagroep et al. [36]	Argentina	Retrospective, observational	1047	Cu-IUD, CuT380A or Cu-T375
Scavuzzi et al. [37]	Brazil	Cross-sectional, nulligravida vs parous women	157	Cu-IUD, CuT380A
Wiebe and Trussell [38]	Canada	Prospective case series	51	Cu-IUD, CuT380A
Garbers et al. [39]	USA	Retrospective cohort analysis	283	Cu-IUD, CuT380A
Shimoni et al. 2011[40]	USA	RCT	156	Cu-IUD, immediate vs late insertion following MTPO
Reeves et al. [41]	USA	RCT	198 vs 100	Two Cu-IUDs: VeraCept175 vs CuT380S
Akintomide et al. [42]	UK	Retrospective, comparative, case control review	63 vs 67	Two Cu-IUDs: Mini TT380 Slimline vs standard-sized TT380 Slimline
*LNG-IUD vs Cu-IUD*				
Bachofner et al. [43]	Switzerland	Retrospective comparative chart review	419 vs 296 vs 40	LNG-IUD, 52 mg vs Cu-T IUD (3rd generation) vs GyneFix 300 Cu-IUD
Phillips et al. [44]	USA	Retrospective, comparative, observational	770 vs 186	LNG-IUD vs Cu-IUD
Hall and Kutler [45]	USA	Prospective, comparative, survey	88 vs 21	LNG-IUD (Mirena) vs CuT380A
Maguire et al. [46]	USA	Secondary analysis of RCT assessing lidocaine for insertion pain	62 vs 137	LNG-IUD vs CuT380A
Wildemeersch et al. [47]	Belgium	Analysis of data collected from studies of FibroPlant and GyneFix	104 vs 50	Cu-IUD (GyneFix) vs LNG-IUD (FibroPlant)
Flamant et al. [48]	France	Prospective, comparative, observational	94 vs 43	Cu-IUD vs LNG-IUD
McNicholas et al. [49]	USA	Retrospective, comparative, observational	53 vs 24	LNG-IUD vs Cu-IUD
Lara-Torre et al. [50]	USA	Retrospective, comparative, chart review	77 vs 12	LNG-IUD vs Cu-IUD
*LNG-IUD and/or Cu-IUD vs Implant*				
Piva et al. [51]	Italy	Prospective, comparative, observational	47 vs 6 vs 36	LNG-IUD and Cu-IUD vs implant
Agostini et al. [52]	France	Retrospective, comparative, cross-sectional	5405 vs 3896 vs 1482	LNG-IUD vs Cu-IUD vs ENG implant
Sanders et al. [53]	USA	Prospective, comparative, observational	82 vs 33 vs 65	LNG-IUD (52 mg) vs Cu-IUD (T380) vs ENG implant
Apter et al. [54]	Australia, Finland, France, Norway, Sweden and UK	RCT	382 vs 381	LNG-IUD (Jaydess, 13.5 mg) vs ENG implant
Diedrich et al. [21]	USA	Prospective, comparative, cohort study, Contraceptive CHOICE Project	3001 vs 826 1184	LNG-IUD vs Cu-IUD (T380A) vs ENG
Grunloh et al. [22]	USA	Prospective, comparative, cohort study, Contraceptive CHOICE Project	3610 vs 952 vs 1366	LNG-IUD vs Cu-IUD vs ENG
O'Neil-Callahan et al. [23]	USA	Prospective, comparative, cohort study, Contraceptive CHOICE Project	6153 overall	LNG-IUD vs Cu-IUD vs ENG
Peipert et al. [24]	USA	Prospective, comparative, cohort study, Contraceptive CHOICE Project	1890 vs 434 vs 522	LNG-IUD vs Cu-IUD vs implant (vs non-LARC)
Modesto et al. [55]	Brazil	RCT of routine vs intensive counselling	99 vs 100 vs 98	LNG-IUD vs Cu-IUD (T380A) vs ENG
Short et al. [56]	Multinational	Prospective, comparative, observational	247 vs 116	LNG-IUD (Mirena) vs ENG
Weisberg et al. [57]	Australia	Prospective, comparative, observational	179 vs 132	LNG-IUD (Mirena) vs ENG
Short et al. [58]	Multinational	Prospective, comparative, observational	211 vs 100	LNG-IUD (Mirena) vs ENG

Cu, copper; ENG, etonogestrel; IUD, intrauterine device; IUS, intrauterine system; LARC, long-acting reversible contraceptive; LNG, levonorgestrel; MTOP, medical termination of pregnancy; RCT, randomised clinical trial

Shading indicates publications reporting the results from the same study

^a^n = 1714 successful placement

^b^These references describe the same study with one reporting the results for women undergoing MTOP at ≤ 63, 64–84 and 85–140 days gestation and one including only the second two subgroups

^c^Report different endpoints from the same study

^d^Report data from the same RCT

Twelve studies (18 publications) report data for LNG-IUDs, either from single-arm studies, studies comparing the timing of placement of the device (three studies) or a study comparing two LNG dose levels [11–20, 25–32]. Ten studies (10 publications) report data for Cu-IUDs, including two comparing two different devices [33–42]. A further eight publications describe the results of eight studies comparing an LNG-IUD with a Cu-IUD [43–50]. Twelve publications (describing 12 studies) were identified that reported comparative results for an LNG-IUD versus the etonogestrel-releasing subdermal implant (ENG) [21–24, 51–58]. No studies were identified comparing a Cu-IUD with ENG. Most were prospective (31 studies), including RCTs and prospective observational studies; 9 studies were retrospective and there were 2 cross-sectional studies.

### Bleeding patterns

Eighteen publications (16 studies) reported on bleeding patterns in women following insertion of an LNG-IUD or Cu-IUD [11, 13–16, 18, 21, 25, 26, 28, 29, 32–35, 45, 49, 55]. Various means were used to enquire about bleeding patterns, including asking women to complete a daily bleeding diary, interviews at periodic study visits and completion of a questionnaire during study visits that included questions regarding bleeding patterns. Questionnaires and daily diaries included descriptions of bleeding patterns based on 3–5 levels of bleeding intensity.

Of the publications describing studies which included an LNG-IUD (n = 31), 11 (9 studies) report on bleeding patterns in women following insertion of the device [11, 13–16, 18, 25, 26, 28, 29, 32]. All consistently report a reduction in bleeding in most women, with some reporting amenorrhoea. Furthermore, all studies report reductions in bleeding and increases in the proportion of women with amenorrhoea over time. Cristobal et al. [29] found that 91% of women experienced a reduction in bleeding at 12 months after insertion of an LNG-IUD and 97% reported very limited bleeding at this time point. Carvalho et al. [28] observed that 36% of women reported amenorrhoea at the first visit (at least 2 months after having device placement) and this increased to 55% a year later. Only 7% and 14% of women at the two time points reported having regular menstruation each month. Achieving amenorrhoea and less bleeding were both associated with satisfaction. Darney et al. [11] reported increases in amenorrhoea rates over the first 9 months from 0.4% after 3 months to 19% at 9 months and this then remained the same at 12 months, while Schreiber et al. [13], reporting data for the same study, described increases from 0.4% at 3 months to 36% at the fourth quarter of the third year. Two further studies report on follow-up to 5 years after insertion of the LNG-IUD and observed rates of amenorrhoea in the fifth year of 42% [14] and 62% [32] respectively, with a further increase to 80% seen in the latter study for women using the device for over 5 years. Indeed, the latter study reported a negative correlation between duration of use and bleeding amount. A further study showed decreased bleeding over time following insertion of a subsequent IUD after 4–5 years [15].

Of the studies including Cu-IUDs (n = 26), only three specifically reported on bleeding patterns [33–35]. Yaron et al. [33] reported that 42% of women had heavy blood flow and 56% had moderate blood flow using the Ballerine MIDI IUD; and according to Bateson et al. [35], 43% of women were bothered by heavy bleeding and 35% reported being bothered by prolonged bleeding after using a T-framed Cu-IUD for 12 months. A further study reported a reduction in post-placement bleeding over the first 5 months from insertion of the CuT380A, as assessed using the Pictorial Blood Assessment Chart [34].

Four of the studies comparing LNG-IUDs with Cu-IUDs report differences in the bleeding patterns between the two types of device [21, 45, 49, 55]. Hall and Kutler [45] highlight the difference in bleeding patterns between the LNG-IUD and CuT380A by reporting bleeding symptoms at 6 months after insertion. At this time point, approximately a third of LNG-IUD users reported amenorrhoea and a third reported scant menstrual bleeding, whereas most (> 80%) women using the CuT380A reported heavy bleeding. Differences were also noted in the duration of bleeding with > 90% of the CuT380A group reporting bleeding lasting for ≥ 5 days compared with < 20% of the LNG-IUD group. A second study observed that 77% of women using the LNG-IUD reported lighter bleeding than experienced prior to use of the device compared with 4% of those using the Cu-IUD; furthermore, 67% of the latter group reported having heavier bleeding compared with before they started using the device (compared with 4% of the LNG-IUD group) [49]. Modesto et al. [55] reported that during months 9–12, almost all women using a CuT380A had normal bleeding (relative to baseline) compared with approximately a third using an LNG-IUD. A further study found that 61% of LNG-IUD users versus 25% of Cu-IUD users reported lighter bleeding at 6 months compared with at 3 months, and 25% of LNG-IUD users compared with 15% of Cu-IUD users reported a reduction in the frequency of bleeding between these time points [21].

### Rates of discontinuation

Rates of discontinuation overall or for bleeding were reported in 18 publications (14 studies) for women using LNG-IUDs [11–15, 17–19, 25–27, 29–31, 54, 56–58], 10 publications (10 studies) for women using Cu-IUDs [33–42] and 14 publications (14 studies) reporting comparative data for the two types of IUDs [22–24, 43–50, 52, 53, 55] (see Table 2).

**Table 2:** Summary of overall discontinuation ra﻿tes and rates of discontinuation due to bleeding

References	Study design	LARC	Patients, N	Time period, months	Any discontinuation	Removal	Discontinuation due to bleeding	Discontinuation due to bleeding as % of discontinuations, %
*LNG-IUD*								
Shimoni et al. [25]	Prospective comparative observational study	LNG-IUD, 13.5 mg (Skyla) early vs late menstrual cycle insertion	132	3	–	Removal, 7 (4%)	1 for spotting (< 1%)	14%
Teal et al. [14]	Single-arm phase III study, ACCESS IUS	LNG-IUD, 52 mg (Liletta)	1751^a^	> 7 years	–	Discontinued for an AE, 322 (18.8%)	39 (2.2%)	12%
Darney et al. [11]	Secondary analysis of phase III study, ACCESS IUS	LNG-IUD, 52 mg (Liletta)	1751^a^	12	–	–	29 (1.7%)	–
Schreiber et al. [13]	Secondary analysis of phase III study, ACCESS IUS	LNG-IUD, 52 mg (Liletta)	1751^a^	36	–	–	35 (2.1%); 20 during months 6–18	–
Eisenberg et al. [12]	Single-arm phase III study, ACCESS IUS	LNG20-IUD, 52 mg (Liletta)	1751^a^	36	–	Other AEs leading to discontinuation: expulsion, 3.5%; acne, 1.3%; mood swings, 1.3%	1.5%	–
Neri et al. [26]	Prospective single-arm	LNG-IUD, 6 µg/day (Jaydess)	25	12	–	0		
Vaitsiakhovich et al. [27]	Analysis of data from an observational study and RCT	LNG-IUD, 52 mg (Mirena)	1860	12, 24	12 months, 13.2% 24 months, 21.5%	–	NR	–
Korjamo et al. [20]	RCT	LNG-IUD (Mirena) immediate vs late insertion following MTOP	267	12	Immediate: 20 (15.0%) Late: 43 (32.8%)	Immediate: 10 (7.5%) Late: 15 (11.5%)	NR	–
Cristobal et al. [29]	Prospective, observational, single-arm	LNG-IUD, 52 mg	201	12	Any discontinuations, 5 (2.5%)	–	1 (< 1%) due to bleeding between periods	20%
Whitaker et al. [30]	RCT	LNG-IUD, immediate vs late insertion following caesarean delivery	42	6, 12	6 months Immediate: 30.0% Delayed: 40.9% 12 months Immediate: 40.0% Delayed: 59.1%	–	NR	–
Gemzell-Danielsson et al. [15]	Prospective single-arm	LNG-IUD	204	6, 12	–	Any discontinuations due to AEs, 5 (2.5%)	1 (0.5%)	20%
Armitage et al. [31]	Prospective, observational	LNG-IUD	100 (89 at follow-up)	12	14 (15.7%)	Removal, 10 (9%)	2 (2.2%)	14%
Nelson et al. [18]	RCT	LNG-IUD 13.5 mg vs 19.5 mg	1432 vs 1452	36	43% vs 40%	Discontinuation for AEs, 21.9% vs 19.1%	4.7% vs 4.9%	11% vs 12%
Gemzell-Danielsson et al. [17]	Post-hoc analysis of phase III RCT (Nelson et al. 2013)	LNG-IUD 13.5 mg vs 19.5 mg	1432 vs 1452	12, 36	1 year Nulliparous: 21.2% vs 20.2% Parous: 16.9% vs 14.9% 3 years, Nulliparous: 45.7% vs 41.9% Parous: 41.0% vs 38.9%	Discontinuation due to AEs, 3 year, nulliparous, 26.1% vs 20.6% 3 year, parous, 19.2% vs 18.2%	3-year discontinuation Nulliparous: 5.2% vs 5.6% Parous: 4.5% vs 4.4%	26% vs 27% 23% vs 24%
Apter et al. [54]	RCT	LNG-IUD (Jaydess, 13.5 mg) vs ENG implant	382 vs 381	12	74 (19.6%) vs 102 (26.8%)	–	16 (4.2%) vs 44 (11.5%)	22%
Short et al. [56]	Prospective, comparative, observational	LNG-IUD (Mirena) vs ENG	247 vs 116	24	32 (13%) vs 20 (17%)	–	9 (4%) vs 13 (11%)	28%
Weisberg et al. [57]	Prospective, comparative, observational	LNG-IUD (Mirena) vs ENG	179 vs 132	36	84 (47%) vs 36 (27%)	–	9 (23%) vs 27 (54%)	11%
Short et al. 2012[58]	Prospective, comparative, observational	LNG-IUD (Mirena) vs ENG	211 vs 100	12	12 (6%) vs 11 (11%)	–	6 (3%) vs 9 (9%)	50%
*Cu-IUD*								
Yaron et al. [33]	Retrospective, observational	Cu-IUD, Ballerine MIDI	207	≥ 12	–	Any removal, 56 (27.1%) Any removal excluding for pregnancy, 22.7%	33 (15.9%)	59%
Sanders et al. [34]	Prospective, longitudinal, observational	Cu-IUD, CuT380A	77 (72 at follow-up)	6	–	Any removals, 8 (11%)	NR	–
Bateson et al. [35]	Prospective, observational	Cu-IUD (TT380 short or long, or a Multiload device)	211	12, 36	Any discontinuation 1 year: 20.1% 3 years: 80, 38.7%	For AEs at 3 years, 59 (27.9%)	3-years, 28 (13.3%)	35%
Jagroep et al. [36]	Retrospective, observational	Cu-IUD, Cu-T380A or Cu­T375	1047	5 years	–	Any removal, 188 (18%)	23 (2.2%) due to complications such as pelvic pain or bleeding	12%
Scavuzzi et al. [37]	Cross-sectional, nulligravida vs parous women	Cu-IUD, CuT380A	157	NR	Any discontinuation Nulligravida: 24.1% Parous: 13.4%	–	Nulligravida: 6.0% Parous: 1.4%	25% vs 12%
Wiebe and Trussell [38]	Prospective case series	Cu-IUD, SCu380A	51	12	–	Any removal, 9 (17.6%)	8 (16%) removed for symptoms	–
Garbers et al. [39]	Retrospective cohort analysis	Cu-IUD, CuT380A	283	6, 18	–	Any removal, 6 months, 31 (11%) 18 months, 78 (28%)	18 months, 24 (8.5%)	31%
Shimoni et al. [40]	RCT	Cu-IUD, immediate vs late insertion following MTOP	156	6	–	Any removal Immediate, 10 (14%) Delayed, 5 (8%)	Bleeding and pain cited as main reasons for removal	–
Reeves et al. [41]	RCT	Two Cu-IUDs: VeraCept175 vs CuT380S	198 vs 100	12, 24	Any discontinuation 12 months: 16% vs 32% 24 months: 31% vs 40%	–	For pain/bleeding At 12 months: 3.5% vs 17.0% At 24 months, 3.0% vs 15.1%	22% vs 53% 10% vs 38%
Akintomide et al. [42]	Retrospective, comparative, case control review	Two Cu-IUDs: Mini TT380 Slimline vs standard-sized TT380 Slimline	63 vs 67	12	10 (15%) vs 20 (32%)	–	For pain and bleeding, 3 (4.5%) vs 14 (22%)	30% vs 70%
*LNG-IUD vs Cu-IUD*								
Bachofner et al. [43]	Retrospective comparative chart review	LNG-IUD, 52 mg vs Cu-T IUD (3rd generation) vs GyneFix 300 Cu-IUD 3rd generation Cu-IUDs (Multiload Cu375, Nova-T 380 and Mona Lisa Cu375)	419 vs 296	12, 36	–	Removal 12 months: 77 (18.4%) vs 61 (20.6%) 36 months, 116 (27.7%) vs 98 (33.1%)	12 months: 8 (1.9%) vs 9 (3.0%)	10% vs 15%
Phillips et al. [44]	Retrospective, comparative, observational	LNG-IUD vs Cu-IUD	770 vs 186	24, 36, 48, 60	Any discontinuations 24 months: 35.1% vs 42.3% At any time: 554 (71.9%) vs 100 (53.8%)	–	At any time: 31 (4.0%) vs 18 (9.7%)	6% vs 18%
Hall and Kutler [45]	Prospective, comparative, survey	LNG-IUD (Mirena) vs CuT380A	88 vs 21	12	Any discontinuations, 4 (4.5%) vs 3 (14.3%)	–	0 (0%) vs 2 (9.5%)	0% vs 67%
Maguire et al. [46]	Secondary analysis of RCT assessing lidocaine for insertion pain	LNG-IUD vs CuT380A	62 vs 137	12	–	Removals: 6 (9.7%) vs 15 (10.9%)	–	–
Wildemeersch et al. [47]	Analysis of data collected from studies of FibroPlant and GyneFix	LNG-IUD (FibroPlant) vs Cu-IUD (GyneFix)	50 vs 104	12	Any discontinuation: 2 (4.3%) vs 4 (3.3%)	–	NR	–
Flamant et al. [48]	Prospective, comparative, observational	LNG-IUD vs Cu-IUD	43 vs 94	6	Any discontinuation: 15 (20%) vs 34 (22.1%)	–	1 (2.3%) vs 9 (9.6%)	12% vs 26%
McNicholas et al. [49]	Retrospective, comparative, observational	LNG-IUD vs Cu-IUD	53 vs 24	Median of 9 months	Any discontinuation: 20.8% vs 16.7%	–	NR	–
Lara-Torre et al. [50]	Retrospective, comparative, chart review	LNG-IUD vs Cu-IUD	77 vs 12	36		Removal, 25 (32.6%) vs 7 (58.3%)	For AEs, 17 (22.1%) vs 5 (41.7%)	
*LNG-IUD vs Cu-IUD vs implant*								
Agostini et al. [52]	Retrospective, comparative, cross-sectional	LNG-IUD vs Cu-IUD vs ENG implant	5405 vs 3896 vs 1482	12, 24	12 months: 5.0% vs 5.9% vs 10.6% 24 months: 8.9% vs 11.9% vs 16.4%	–	NR	–
Sanders et al. [53]	Prospective, comparative, observational	LNG-IUD (52 mg) vs Cu-IUD (T380) vs ENG implant	82 vs 33 vs 65	12	10% vs 12% vs 9%	–	NR	–
Grunloh et al. [22]	Prospective, comparative, cohort study, Contraceptive CHOICE Project	LNG-IUD vs Cu-IUD vs ENG	3610 vs 952 vs 1366	6	263 (7.3%) vs 76 (8.0%) vs 94 (6.9%)	–	Heavy bleeding: 3 (0.1%) vs 9 (0.9%) vs 0 Irregular/frequent bleeding: 14 (0.4%) vs 10 (1.1%) vs 50 (3.7%)	1% vs 11% vs 0%
O'Neil-Callahan et al. [23]	Prospective, comparative, cohort study, Contraceptive CHOICE Project	LNG-IUD vs Cu-IUD vs ENG	4423 (LARC)	12, 24	12 months: 12% vs 15% vs 17% 24 months: 21% vs 23% vs 31%	–	NR	–
Peipert et al. [24]	Prospective, comparative, cohort study, Contraceptive CHOICE Project	LNG-IUD vs Cu-IUD vs implant (vs non-LARC)	1890 vs 434 vs 522	12	12.5% vs 16.0% vs 16.7%	–	For bleeding or cramps, 5% vs 14% vs 10%	–
Modesto et al. [55]	RCT of routine vs intensive counselling	LNG-IUD vs CuT380A IUD vs ENG	99 vs 100 vs 98	12	19% vs 26.8% vs 17.4%	–	2.7% vs 4.0% vs 2.1%	14% vs 15% vs 12%

AE, adverse event; Cu, copper; ENG, etonogestrel; IUD, intrauterine device; IUD, intrauterine device; LARC, long-acting reversible contraceptive; LNG, levonorgestrel; MTOP, medical termination of pregnancy; NR, not reported; RCT, randomised clinical trial

Shading indicates publications reporting the results from the same study

^a^n = 1714 successful placement

#### LNG-IUD

Rates of discontinuation for any reason in studies of LNG-IUDs were reported in 11 publications (10 studies, including 4 that compared LNG-IUDs with ENG, but excluding those compared with Cu-IUDs) [17, 18, 20, 27, 29–31, 54, 56–58]. In seven of the studies, 12-month rates of discontinuation reported in individual studies ranged between 13 and 21% [17, 20, 27, 31, 54, 56], whereas lower 12-month rates were reported in a single-arm prospective observational study (2.5%) [29], and a prospective observational study comparing an LNG-IUD with ENG (6%) [58]. A small RCT (n = 42) comparing immediate (intra-caesarean) versus routine postpartum placement reported rates of 40% and 59%, respectively, with the higher rate in women who had immediate placement reflecting a rate of expulsion of 20% [30]. Two studies reported discontinuation rates at 36 months with rates being 40% and 47%, respectively [18, 57].

Three further publications reported rates of discontinuation due to adverse events (AEs) and these ranged from 2.5% at 12 months in a prospective study performed in four European countries [15] to approximately 20% at 12 months in a large multinational RCT comparing two LNG-IUD doses [18]. The rate was 19% at 7 years in an analysis of long-term follow-up data from the large (n = 1751) single-arm phase III trial of LNG-IUS, ACCESS [14].

Fourteen publications (10 studies) reported the rates of discontinuation due to bleeding (which in some studies could include amenorrhoea) [11–15, 17, 18, 25, 29, 31, 54, 56–58]. Values from individual studies ranged from < 1% at 3 or 12 months in three studies [15, 25, 29] to approximately 2% in the ACCESS trial at (1–7 + years) [11–14] and a prospective observational study [31], and was approximately 5% at 3 years in an RCT comparing two doses of LNG-IUD [17, 18]. In four studies comparing LNG-IUD with implants, discontinuation rates due to bleeding in the individual studies ranged between 3 and 4% at 12 months (three studies) [54, 56, 58] and was 23% at 36 months in the fourth study [57]. In most studies where reasons were reported, bleeding concerns were a minor proportion (11–30%) of the total cases of discontinuation [14, 15, 17, 25, 29, 31, 54, 56, 57]. However, in one study, half of the discontinuations (in 6% of patients due to any cause) were because of bleeding [58].

#### Cu-IUD

Ten publications (10 studies) reported rates of discontinuation/removal of Cu-IUDs for any reason [33–42], with rates from individual studies ranging from approximately 10% at 6 months in two studies [34, 40] to approximately 16–32% at 1 year in five studies [33, 35, 38, 41, 42] and 40% at 2 years and 3 years in two studies [35, 41]. Lower rates were reported for the VeraCept175 and Mini TT380 Slimline in two comparative studies, with 12-month rates being 16% [41] and 15% [42], respectively, for these Cu-IUDs compared with 32% for CuT380A. Rates of discontinuation due to bleeding were reported in eight studies [33, 35–39, 41, 42]. Rates were < 5% in a large 5-year retrospective study [36], a cross-sectional study performed in Brazil [37] and for the smaller Cu-IUDs, VeraCept175 and Mini TT380 Slimline [41, 42], whereas in other studies (including for the comparator group in the studies of VerCept175 and MiniTT380) the reported rates for individual studies ranged from 8.5 to 22%. Thus, in most studies, bleeding (and pain) was the reason for discontinuation in over a third of women choosing to have Cu-IUDs removed.

#### LNG-IUD versus Cu-IUD

**Fig. 2:**
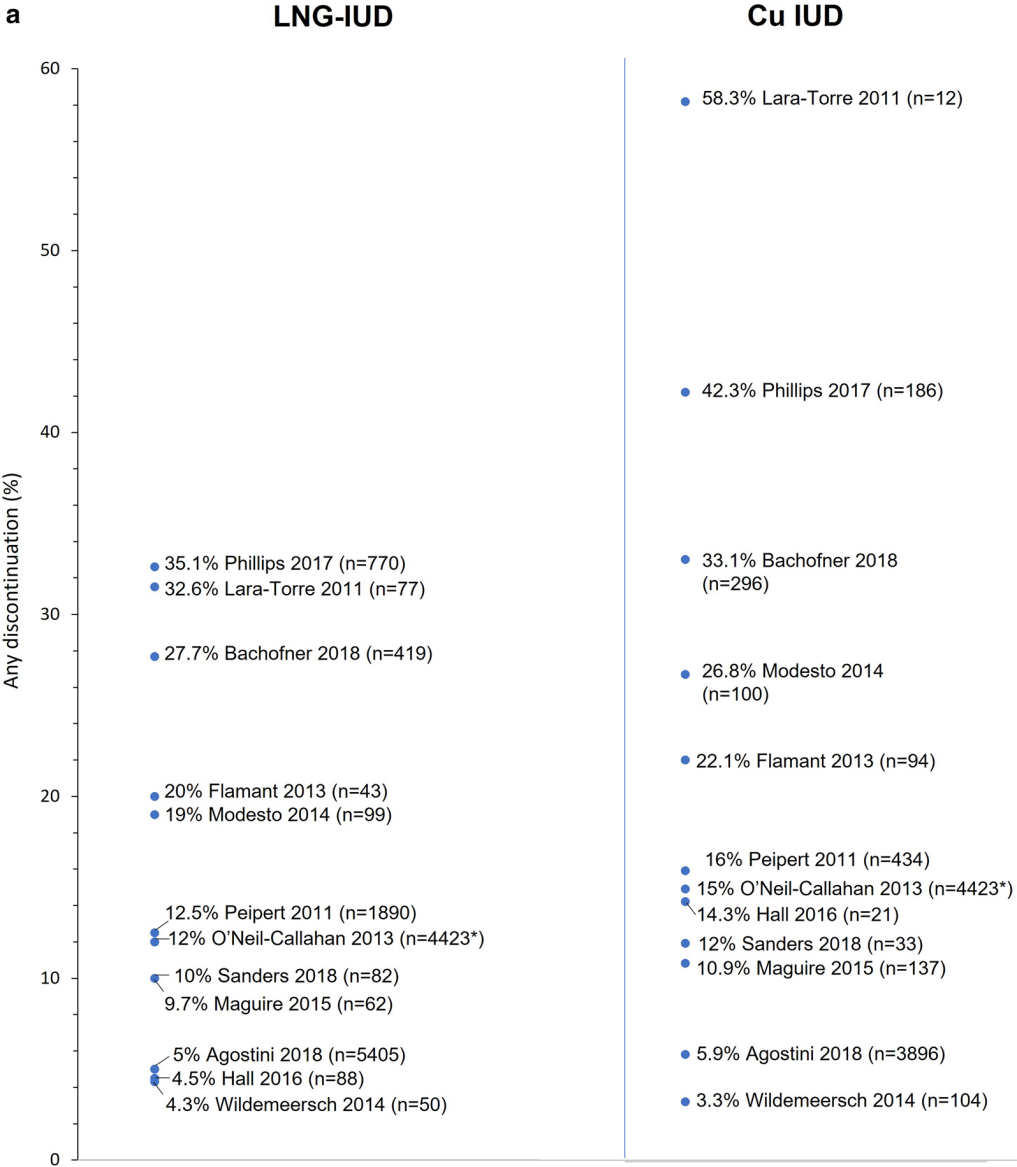

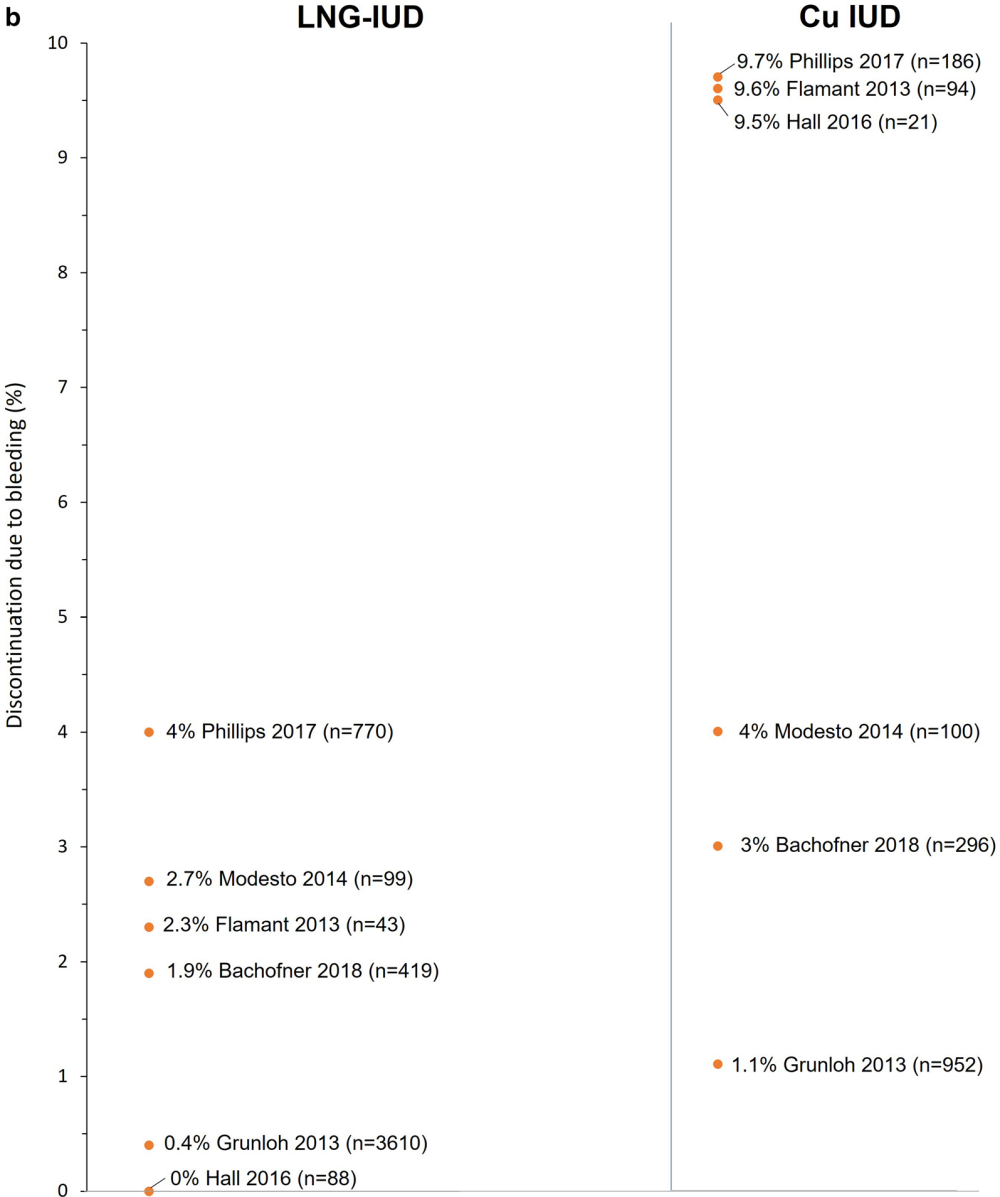
Rates of discontinuation for any reason 12–36 months after insertion (**a**) and discontinuation for bleeding at any time (**b**) for studies reporting data for levonorgestrel-releasing intrauterine systems (LNG-IUDs) and copper intrauterine devices (Cu-IUDs). *N for LNG-IUD and Cu-IUD

Fourteen comparative studies (14 publications) report rates of discontinuation/removal for women using LNG-IUDs versus Cu-IUDs (four of which also compared IUCs with ENG) [22–24, 43–50, 52, 53, 55]. Nine of the 11 studies that reported rates of discontinuation for any reason reported higher rates for Cu-IUDs versus LNG-IUDs as did all three of the studies which reported rates of removal (for any reason). Thus, rates of discontinuation at 12 months in individual studies ranged from 4.3 to 19% for LNG-IUDs and from 3.3 to 26.8% for Cu-IUDs (Fig. 2). Similarly, a large retrospective observational study [44] reporting rates of discontinuation at 24 months observed a lower rate of 35.1% for LNG-IUDs compared with 42.3% for Cu-IUDs, while two studies reporting rates of removal by 36 months also reported higher rates for Cu-IUDs versus LNG-IUDs (33.1% vs 27.7% and 58.3% vs 32.6%) [43, 50].

Rates of discontinuation due to bleeding were reported in six studies and, in all but one, rates were at least two-fold higher for Cu-IUDs versus LNG-IUDs (range of values across the six studies: LNG-IUD, 0.1–4.0%; Cu-IUDs, 1.1–14.3%) [22, 43–45, 48, 55]. Thus, bleeding accounted for 1–14% of discontinuations in women using the LNG-IUD in five of the six studies versus 11–26% of discontinuations in those using a Cu-IUD [22, 43, 44, 48, 55]. The sixth study involved 109 patients, 7 of whom discontinued by 12 months, with bleeding accounting for 2 of the 3 patients who discontinued the Cu-IUD (out of 21 using this device) and none of 4 patients who discontinued the LNG-IUD (out of a total of 88 patients with an LNG-IUD) [45]. Removals for bleeding accounted for 10% of removals by 12 months in LNG-IUD users and 15% of Cu-IUD users in the large retrospective observational study described by Bachofner et al. [43].

### Satisfaction ratings

Nineteen publications (18 studies) report satisfaction ratings for the LNG-IUD or Cu-IUD [16–18, 21, 24, 26, 28, 30, 32, 33, 37, 38, 48, 49, 51, 54–56, 58] (Table 3). Nine studies (10 publications) of LNG-IUD included assessments of overall satisfaction with the device [16–18, 26, 28, 30, 32, 54, 56, 58]. Of these, six studies (seven publications) reported that > 90% of women were satisfied (somewhat/moderate or very/highly satisfied) with the device [16–18, 26, 28, 30, 32] and the other three studies reported 80–87% of women (across the three studies) to be satisfied [54, 56, 58]. Five studies (six publications) also assessed satisfaction with bleeding and reported 61–92% of women being satisfied (somewhat/very satisfied) [16, 18, 32, 54, 56] [17]. Three studies of Cu-IUDs reported overall satisfaction ratings. These ranged from 66 to 95% [33, 37, 38]. None of these studies included assessments of satisfaction with bleeding. Six studies provide comparative satisfaction rates for LNG-IUDs versus Cu-IUDs [21, 24, 48, 49, 51, 55]. No studies reported statistically significant differences between the two types of IUC. Satisfaction ratings ranged from 79 to 94% for LNG-IUDs and 80–100% for Cu-IUDs.

**Table 3:** Summary of satisfaction ratings

References	Study design	LARC	Time period (months)	Patient satisfaction with contraception, %	Satisfaction with bleeding, %
*LNG-IUD*					
Neri et al. [26]	Prospective single-arm	LNG-IUD, 6 µg/day (Jaydess)	12	100% (excellent/optimal/good)	–
Carvalho et al. [28]	Prospective, observational, single-arm	LNG-IUD, 20 µg/day (Mirena)	> 14	93% highly satisfied	–
Whitaker et al. [30]	RCT	LNG-IUD, immediate vs late insertion following caesarean delivery	12	Immediate vs delayed 91.7% vs 100% (with available data)	–
Stoegerer-Hecher et al. [32]	Cross-sectional	LNG-IUD (Mirena)	NR	90.6% (very/quite/moderately satisfied)	74.1% very satisfied amenorrhoeic, 91.0%
Heikinheimo et al. [16]	Prospective, single-arm	LNG-IUD	12	98.4% (definite/somewhat agreeing)	91.7% (definite/somewhat agreeing)
Nelson et al. [18]^a^	RCT	LNG-IUD 13.5 mg vs 19.5 mg	36	95% vs 96% (very/somewhat satisfied)	77% vs 76% (very/somewhat satisfied)
Gemzell-Danielsson et al. [17]^a^	RCT (same study as Nelson et al. 2013)	LNG-IUD 13.5 mg vs 19.5 mg	36	> 90% (very/somewhat satisfied)	> 70% (very/somewhat satisfied)
Apter et al. [54]	RCT	LNG-IUD (Jaydess, 13.5 mg) vs ENG	12	86.5% vs 75.9% (very/somewhat satisfied)	60.9% vs 33.6% (very/somewhat satisfied)
Short et al. [56]	Prospective	LNG-IUD (Mirena) vs ENG	24	84% vs 70% (agree)	90% vs 77% (agree)
Short et al. [58]	Prospective, comparative, observational	LNG-IUD (Mirena) vs ENG	12	80% vs 66% (definite/somewhat agree)	–
*Cu-IUD*					
Yaron et al. [33]	Retrospective, observational	Cu-IUD, Ballerine MIDI	–	65.7% satisfied/very satisfied	–
Scavuzzi et al. [37]	Cross-sectional, nulligravida vs parous women	Cu-IUD, CuT380A	–	Nulligravida/parous 93.8% vs 94.5% (fully/partially satisfied)	–
Wiebe and Trussell [38]	Prospective case series	Cu-IUD, SCu380A	12	71% satisfied	–
*LNG-IUD vs Cu-IUD*					
Flamant et al. [48]	Prospective, comparative, observational	LNG-IUDvs Cu-IUD	6	82.1% vs 86.7% (very/somewhat satisfied) (*p* = 0.81)	–
McNicholas et al. [49]	Retrospective, comparative, observational	LNG-IUD vs Cu-IUD	9	78.7% vs 85.0% (satisfied) (*p* = 0.99)	–
Piva et al. [51]	Prospective, comparative, observational	LNG-IUD vs Cu-IUD vs implant	12	87.2% vs 100% vs 63.4%, ns (ITT analysis)	–
Diedrich et al. [21]	Prospective, comparative, cohort study, Contraceptive CHOICE Project	LNG-IUD vs Cu-IUD (CuT380A) vs ENG	6	94% vs 93% vs 90% (very/somewhat satisfied)	–
Modesto et al. [55]	RCT of routine vs intensive counselling	LNG-IUD vs CuT380A IUD vs ENG	12	91.0% vs 85.7% vs 90.0% (*p* = 0.612)	–
Peipert et al. [24]	Prospective, comparative, cohort study, Contraceptive CHOICE Project	LNG-IUD vs Cu-IUD vs Implant (vs non-LARC)	12	85.7% vs 80.1% vs 78.7% (very/somewhat satisfied)	–

Cu, copper; ENG, etonogestrel; IUD, intrauterine device; IUS, intrauterine system; ITT, intention-to-treat; LARC, long-acting reversible contraceptive; LNG, levonorgestrel; NR, not reported; ns, not significant; RCT, randomised clinical trial

Shading indicates publications reporting the results from the same study

^a^Report data from the same RCT

## Discussion

### Findings and interpretation

Bleeding changes resulting from contraceptive use are an important contributor to uptake and continuation of all contraceptive methods, including IUCs. However, limitations in reporting, differences in study populations and patient preferences make it difficult to set individual expectations about bleeding. We sought to determine a broader perspective on how bleeding affects discontinuation of IUCs, based on all available data from recent studies in relevant populations, as this can represent a useful clinical endpoint for patients who are deciding whether or not intrauterine contraception is right for them.

The findings from this systematic literature review suggest that the difference in bleeding profiles between LNG-IUDs and Cu-IUDs may account for some of the differences in discontinuation rates between the two types of device. This is based on a review of 48 publications (describing 42 studies) reporting on bleeding, discontinuation rates and/or satisfaction rates in women using LNG-IUDs or Cu-IUDs published over a 10-year period and performed in North America, Europe, South America or Australia.

Firstly, the review found that studies reporting on the bleeding profile following insertion of either type of device indicate that many LNG-IUD users experience a reduction in bleeding, with further reductions occurring over time and many women becoming amenorrhoeic. Indeed, approximately 90% of users experience a reduction in bleeding at 1 year with an LNG-IUD and most report further reductions in bleeding compared with their prior visit at 3, 6 and 12 months. In contrast, two studies reporting on bleeding in users of Cu-IUDs observed that approximately 40% of women experienced heavy bleeding or were bothered by prolonged bleeding. These differences were clearly demonstrated in five comparative studies reporting on bleeding profile according to type of IUC.

Secondly, the findings highlight the importance of the difference in bleeding profile to users, as illustrated by the discontinuation rates reported for LNG-IUDs and Cu-IUDs in 42 publications (38 studies) reporting rates of continuation/discontinuation or removal for either type of device. Most comparative studies reported higher rates of discontinuation for Cu-IUDs compared with LNG-IUDs. Although most studies were not designed to compare discontinuation rates and did not report whether differences were statistically significant, the overall trend seen across studies suggests that discontinuation rates are higher for Cu-IUDs and this is further supported by rates of discontinuation reported for either type of IUC in single-arm studies. Importantly, most studies (26 of 28) were observational or cross-sectional and are thus more likely to reflect real-world experience rather than being influenced by use in a trial setting.

Twenty-eight publications (24 studies) reported rates of discontinuation due to bleeding profile [11–15, 17, 18, 22, 25, 29, 31, 33, 35–39, 41–45, 48, 54–58]. In LNG-IUD users, discontinuation for bleeding was generally low and constituted less than a third of cases of discontinuation. In contrast, rates of discontinuation for bleeding tended to be higher with Cu-IUDs and bleeding was the reason for discontinuation in up to 26% of women discontinuing their Cu-IUD. This trend is supported by comparative studies reporting rates of discontinuation overall and due to bleeding for women using LNG-IUDs versus Cu-IUDs. Reassuringly, discontinuation due to bleeding appears to be a short-term (within 1 year or less) phenomenon, with relatively few women requesting removal in longer-term studies after the first year of use. Comparison of rates of discontinuation for any reason and for bleeding suggest that the latter is a significant cause of discontinuation in Cu-IUD users.

Understanding factors that contribute to a woman’s decision to continue or discontinue use of an IUC could help women in their choice of contraception and type of IUC [59]. Few studies have specifically compared continuation/discontinuation rates for LNG-IUDs and Cu-IUDs or the role of bleeding profile in a women’s decision to request removal of the device, as revealed by the published literature identified in this systematic literature review. However, this review has revealed that there is a substantial body of published evidence reporting overall discontinuation rates and discontinuation rates due to bleeding that can be used to guide women and their physicians in the choice of contraception.

Discontinuation serves as both an objective endpoint and a clinically relevant one for women and their providers. In contrast, satisfaction with a contraceptive method is less precise, more subjective and highly individualised. Despite significant differences in bleeding, satisfaction rates were high in most studies, and rates overlap between LNG-IUD and Cu-IUD users. Providers therefore must work collaboratively with patients to help find the best method for them.

### Strength and weaknesses

This review has a number of limitations that should be considered when interpreting the findings. Firstly, there are few studies directly investigating the relationship between bleeding profile and discontinuation rates; hence most of the relevant data identified were not from comparative or powered studies. Secondly, the studies identified differ widely in many aspects of design; participants; satisfaction ratings; and how bleeding profiles were quantified. Comparisons between studies therefore are made judiciously. This likely explains the wide range of rates of discontinuation observed overall and for bleeding between different studies of the same IUC. Thirdly, this review was limited to studies performed in North and South America, Europe and Australia. Thus, the findings may not be generalisable to other geographical regions. Indeed, it was decided to exclude publications from countries in Asia, the Middle East and Africa because, in many of these cultures, bleeding is seen as being favourable and would be unlikely to be a reason for discontinuing IUD use. Indeed, some studies indicate that contrary to findings from the US and Europe where increased bleeding is often a driver of discontinuation, in countries in Asia it is in fact a lack of bleeding which is cited as a reason for discontinuation [60]. We do not suggest that the findings of this review are relevant for these geographical regions. Similarly, the review was limited to studies published from 2010 onwards as changing attitudes over time mean that results of studies performed before this date may not be generalisable to current practice. Lastly, we intentionally excluded LNG-IUD studies where there was a therapeutic indication, such as heavy menstrual bleeding or fibroids. However, not all studies specifically excluded these participants, and it is likely that some patients chose an LNG-IUD owing to problematic bleeding or desire for reduced bleeding. In contrast, persistence of menses may motivate some Cu-IUD users. The potential for bias due to confounding would result in an over-reporting of bleeding and discontinuation and may account for some studies with unexpectedly high reported rates of these outcomes. Furthermore, women with pre-existing heavy bleeding may be counselled away from a Cu-IUD and toward the LNG-IUD for contraceptive use. Despite these limitations, the study provides substantial evidence for differences in continuation rates for LNG-IUDSs and Cu-IUDs and the influence of bleeding profile on continuation.

### Open questions and future research

The findings of this systematic literature review suggest that bleeding is associated with discontinuation of IUCs, and that the reduction in bleeding experienced by women following insertion of an LNG-IUD is associated with higher continuation rates compared with use of a Cu-IUD. Fewer women discontinue an LNG-IUD due to unfavourable or heavy bleeding when compared with those using a Cu-IUD (or, by comparison, an ENG implant). Among discontinuers, bleeding is a fairly commonly cited reason for removing an IUC. These results reinforce the need for good counselling and expectation-setting around bleeding with an IUC, and the continued search for ways to improve bleeding among IUC and long-acting reversible contraception users. The findings also indicate that interventions to improve early bleeding changes could have a significant impact on continuation of an IUC.

## Supplementary Information

**Additional file 1**. Appendix.

## Data Availability

Not applicable.

## Abbreviations

AE: Adverse event
Cu-IUD: Copper intrauterine device
ENG: Etonogestrel
IUC: Intrauterine contraceptive
IUD: Intrauterine device
LARC: Long-acting reversible contraception
LNG-IUD: Levonorgestrel-releasing intrauterine device
LNG-IUS: Levonorgestrel-releasing intrauterine system
MTOP: Medical termination of pregnancy
NOS: Newcastle–Ottawa Scale
PRISMA-P: Preferred Reporting Items for Systematic Reviews and Meta-Analysis Protocol
RCT: Randomised clinical trial
SLR: Systematic literature review
