# Supplementary material for: Discontinuation rates of intrauterine contraception due to unfavourable bleeding: a systematic review
Source: BMC Womens Health. 2022 Mar 21;22:82. doi: 10.1186/s12905-022-01657-6 (PMC8939098; doi:10.1186/s12905-022-01657-6)
Supplement: Supplementary file 1 — Additional file 1. Appendix. [file 12905_2022_1657_MOESM1_ESM.docx]

Appendix 1

Search strings used in PubMed

| **Parameter** | **# ID** | **Search** | **Hits  (28-Nov-2019)** |
| --- | --- | --- | --- |
| LARCs |  | Long-Acting Reversible Contraception[MeSH Terms] OR contraceptive iud[MeSH Terms] OR copper releasing iud[MeSH Terms] OR hormone releasing iud[MeSH Terms] | 11,444 |
|  |  | "long acting reversible contraceptive"[Title/Abstract] OR "long acting reversible contraceptives"[Title/Abstract] OR "LNG-IUS"[Title/Abstract] OR "LNG-IUDs"[Title/Abstract] | 1194 |
|  |  | "Cu-IUD"[Title/Abstract] OR "copper-IUDs"[Title/Abstract] OR "Cu-T"[Title/Abstract] OR "copper-T"[Title/Abstract] OR gynefix[Title/Abstract] OR TCu380a[Title/Abstract] OR paragard[Title/Abstract] OR SMB[Title/Abstract] | 2121 |
|  |  | (Hormon*[Title/Abstract] OR "hormonal"[Title/Abstract] OR "Copper"[Title/Abstract] OR "Cu"[Title/Abstract] OR "levonorgestrel-releasing"[Title/Abstract] OR "LNG-releasing"[Title/Abstract] OR "LNG"[Title/Abstract]) AND (IUS[Title/Abstract] OR IUD[Title/Abstract] OR IUDs[Title/Abstract] OR IUCD[Title/Abstract] OR IUCDs[Title/Abstract] OR LARC[Title/Abstract] OR LARCs[Title/Abstract] OR "intrauterine system"[Title/Abstract] OR "intrauterine device"[Title/Abstract] OR "intrauterine devices"[Title/Abstract] OR "intrauterine contraceptive device"[Title/Abstract] OR "intrauterine contraceptive devices"[Title/Abstract] OR "intra-uterine system"[Title/Abstract] OR "intra-uterine device"[Title/Abstract] OR "intra-uterine devices"[Title/Abstract] OR "intra-uterine contraceptive device"[Title/Abstract] OR "intra-uterine contraceptive devices"[Title/Abstract] OR levosert[Title/Abstract] OR liletta[Title/Abstract] OR progestasert[Title/Abstract]) | 4953 |
|  |  | ("Lippes Loop"[Title/Abstract] OR "Lippes-Loop"[Title/Abstract] OR antigon[Title/Abstract] OR beospir[Title/Abstract] OR intragral[Title/Abstract] OR birnberg[Title/Abstract] OR butterfly[Title/Abstract] OR "dalkon shield"[Title/Abstract] OR gynekoil[Title/Abstract] OR margulies[Title/Abstract] OR "polish spiran"[Title/Abstract] OR "k s wing"[Title/Abstract] OR "heart iud"[Title/Abstract] OR "hong kong triangle"[Title/Abstract] OR ahmed[Title/Abstract] OR "pleated membrane"[Title/Abstract] OR lem[Title/Abstract] OR "quadricoil"[Title/Abstract] OR corolle[Title/Abstract] OR "saf t coil"[Title/Abstract] OR "saf t"[Title/Abstract] OR "soonawala"[Title/Abstract] OR "spring coil"[Title/Abstract] OR "dana cor"[Title/Abstract] OR dana[Title/Abstract] OR Mirena[Title/Abstract] OR Skyla[Title/Abstract] OR Jaydess[Title/Abstract] OR Kyleena[Title/Abstract] OR LCS[Title/Abstract]) AND (contraceptive[Title/Abstract] OR contraception[Title/Abstract] OR IUS[Title/Abstract] OR IUD[Title/Abstract] OR IUDs[Title/Abstract] OR IUCD[Title/Abstract] OR IUCDs[Title/Abstract] OR LARC[Title/Abstract] OR LARCs[Title/Abstract] OR "intrauterine system"[Title/Abstract] OR "intrauterine device"[Title/Abstract] OR "intrauterine devices"[Title/Abstract] OR "intrauterine contraceptive device"[Title/Abstract] OR "intrauterine contraceptive devices"[Title/Abstract] OR "intra-uterine system"[Title/Abstract] OR "intra-uterine device"[Title/Abstract] OR "intra-uterine devices"[Title/Abstract] OR "intra-uterine contraceptive device"[Title/Abstract] OR "intra-uterine contraceptive devices"[Title/Abstract] OR levosert[Title/Abstract] OR liletta[Title/Abstract] OR progestasert[Title/Abstract]) | 1016 |
|  |  | #1 OR #2 OR #3 OR #4 OR #5 | 14,127 |
| Outcomes |  | continu*[Title/Abstract] OR discontinu*[Title/Abstract] OR "discontinuation rate"[Title/Abstract] OR bleeding[Title/Abstract] OR safe[Title/Abstract] OR safer[Title/Abstract] OR safety[Title/Abstract] OR efficac*[Title/Abstract] OR efficacy[Title/Abstract] OR effective[Title/Abstract] OR efficient[Title/Abstract] OR adverse[Title/Abstract] OR success[Title/Abstract] OR "success-rate"[Title/Abstract] OR failure[Title/Abstract] OR spotting[Title/Abstract] OR menorrhagia[Title/Abstract] OR amenorrhoea[Title/Abstract] OR amenorrhea[Title/Abstract] OR dysmenorrhoea[Title/Abstract] OR dysmenorrhea[Title/Abstract] OR "adverse device effect"[Title/Abstract] OR "device comparison"[Title/Abstract] OR acceptability[Title/Abstract] OR tolera*[Title/Abstract] OR acceptance[Title/Abstract] OR removal[Title/Abstract] | 52,01,555 |
| LARCs and outcomes |  | #6 AND #7 | 6476 |
| Filter: Years |  | (#6 AND #7) Filters: Publication date from 2000/01/01 to 2019/12/31 | 3288 |
|  |  | (#6 AND #7) Filters: Publication date from 2000/01/01 to 2019/12/31; Humans | 2744 |
|  |  | (#6 AND #7) Filters: Publication date from 2000/01/01 to 2019/12/31; Humans; Female | 2699 |

Appendix 2

Eligibility criteria used in the systematic screening strategy to identify publications relating to use and discontinuation rates of intrauterine contraceptives

|  | **Inclusion** | **Exclusion** |
| --- | --- | --- |
| Population | Females >18 years having normal reproductive health using an IUD or having used any IUD in the past | Females on oral contraceptives or sex hormone therapy  Females with comorbidities affecting reproductive health  Females at risk of breast cancer  Women receiving IUDs for indications such as menorrhagia/heavy bleeding or adenomyosis |
| Intervention(s) | Reversible contraceptives (IUDs and IUSs) | Studies involving contraceptive methods other than IUDs |
| Outcomes | Continuation and discontinuation of IUDs and reasons for discontinuation  Contraception success with IUDs/LNG-IUSs  Bleeding profile/pattern  Side-effects observed with IUDs/LNG-IUSs (e.g. menstrual disorders, oedema, headache, breast tenderness, lower abdominal and/or back pain, vaginal discharge, nausea) in the first year after insertion/implantation  Complications with IUD/LNG-IUS use  Impact of discontinuation, e.g. pregnancy  Time to removal/side-effects after insertion  Any relevant patient-reported outcomes, e.g. patient satisfaction, acceptability and tolerability  Studies comparing IUDs or IUSs with other methods of contraception | Studies reporting non-contraceptive health benefits of IUDs |
| Study design/  type of studies | Randomised and non-randomised phase II and phase III clinical trials  USA and Europe Guidelines on IUDs or IUSs  Real-world prospective observational studies (primary data collection) or data-base studies (secondary data, retrospective analyses) | In vitro studies  Studies reporting use of IUDs other than for contraception, such as gynaecological disorders, including menorrhagia, anaemia, hormone replacement therapy and endometrial cancer prevention  Use of sex hormones other than for contraception |
| Publication | Primary publications,  Secondary publications/subgroup analyses,  Pooled analyses | Systematic reviews (flagged)  Narrative reviews (flagged)  Congress abstracts  Case reports  Case series  Letters, editorials, opinion, etc. |
| Language | English | Non-English language |
| Study period | 2000–2019 | Before year 2000 |

IUD, intrauterine device; IUS, intrauterine system LNG, levonorgestrel.

Appendix 3.

Assessment of risk of bias

## Methodology

The quality of the included study was assessed by the Newcastle–Ottawa Scale (NOS). (<http://www.ohri.ca/programs/clinical_epidemiology/oxford.asp>). A total of 29 cohort studies, 17 case-control or RCTs and two cross-sectional studies were subjected to three different sets of quality assessment scales. The NOS questionnaire has three major domains, i.e. 1. Selection, 2. Comparability, 3. Outcomes. A study can be awarded a maximum of one star for each numbered item within the Selection and Outcome categories and a maximum of two stars for Comparability.

**Selection:**

1. Representativeness of exposed cohort: 1, study population truly or somewhat representative of a community/population-based study; 0, study population was sampled from a special population, that is, population from a company, hospital patients, data from the health insurance company or health examination organisation, nurses.
2. Selection of non-exposed cohort: 1, drawn from the same community as the exposed cohort.
3. Ascertainment of exposure: 1, validation of pets use with secure record; 0, no specific pets use validation method.
4. Demonstration that outcome was not present at start of study: 1, exclusion of participants with a history of severe ventricular arrhythmia or sudden cardiac arrest at the beginning of the study.

**Comparability:**

1. Whether a study is adjusted for the most important factors deliberately.
2. Whether a study is adjusted for other important risk factors.

**Outcome:**

1. Assessment of outcome: 1, cardiovascular events were confirmed by medical records or record linkage; 0, self- reported.
2. Was follow-up long enough for outcomes to occur: 1, duration of follow-up ≥5 year; 0, if duration of follow-up < 5 year.
3. Loss to follow-up rate: 1, complete follow-up or loss to follow up rate ≤20 %; 0, follow-up rate < 80% or no description of those lost.

## Results

The results of the risk of bias analysis are tabulated below.

Table A. Overall quality assessment

| **Study design** | **Good** | **Fair/satisfactory** | **Poor** |
| --- | --- | --- | --- |
| Cohort study (29) | 10 | 1 | 18 |
| Case control/RCT (17) | 8 | 0 | 9 |
| Cross-sectional study (2) | 0 | 2 | 0 |

Table B. Detailed Newcastle–Ottawa Scale scores of cohort studies

| **Study** | **Selection** | | | | **Comparability** | | **Outcome** | | |  | |
| --- | --- | --- | --- | --- | --- | --- | --- | --- | --- | --- | --- |
|  | **Represent-ativ eness of exposed cohort** | **Selection of non- exposed cohort** | **Ascertainment of exposure** | **Demonstr-ation that outcome of interest was not present at start of study** | **Adjust for the most import-ant risk factors** | **Adjust for other risk factors** | **Assessment of outcome** | **Follow-up length** | **Loss to follow-up rate** | **Total score (quality)^a^** |  |
| Agostini et al. 2018 [52] | 1 | 1 | 1 | 1 | 0 | 0 | 1 | 1 | 0 | **6 (Poor)** |  |
| Armitage et al. 2013 [31] | 0 | 0 | 0 | 0 | 0 | 0 | 0 | 1 | 1 | **2 (Poor)** |  |
| Bachofner et al. 2018 [43] | 1 | 1 | 1 | 1 | 1 | 1 | 1 | 1 | 1 | **9 (Good)** |  |
| Bateson et al. 2016 [35] | 0 | 0 | 1 | 0 | 1 | 1 | 0 | 1 | 1 | **5 (Poor)** |  |
| Carvalho et al. 2017 [28] | 1 | 0 | 1 | 1 | 1 | 1 | 0 | 1 | 0 | **6 (Poor)** |  |
| Cristobal et al. 2016 [29] | 1 | 0 | 1 | 1 | 1 | 1 | 0 | 1 | 0 | **6 (Poor)** |  |
| Darney et al. 2018 [11] | 1 | 1 | 0 | 1 | 1 | 1 | 0 | 1 | 0 | **6 (Poor)** |  |
| Diedrich et al. 2015 [21] | 1 | 1 | 1 | 1 | 1 | 1 | 0 | 1 | 1 | **8 (Good)** |  |
| Flamant et al. 2013 [48] | 1 | 1 | 0 | 1 | 1 | 1 | 0 | 1 | 1 | **7 (Good)** |  |
| Garbers et al. 2013 [39] | 0 | 1 | 1 | 1 | 1 | 1 | 0 | 1 | 0 | **6 (Poor)** |  |
| Gemzell-Danielsson et al. 2010 [15] | 1 | 1 | 1 | 1 | 0 | 0 | 0 | 1 | 1 | **6 (Poor)** |  |
| Grunloh et al. 2013 [22] | 1 | 1 | 1 | 1 | 1 | 1 | 1 | 1 | 1 | **9 (Good)** |  |
| Hall and Kutler 2016 [45] | 1 | 1 | 0 | 1 | 0 | 0 | 0 | 1 | 1 | **5 (Poor)** |  |
| Heikinheimo et al. 2010 [16] | 1 | 1 | 0 | 1 | 1 | 1 | 0 | 1 | 0 | **6 (Poor)** |  |
| Jagroep et al. 2016 [36] | 1 | 1 | 1 | 1 | 0 | 0 | 1 | 1 | 1 | **7 (Poor)** |  |
| Lara-Torre et al. 2011 [50] | 1 | 0 | 1 | 1 | 0 | 0 | 1 | 1 | 0 | **5 (Poor)** |  |
| McNicholas et al. 2012 [49] | 1 | 1 | 0 | 1 | 1 | 1 | 0 | 0 | 1 | **6 (Poor)** |  |
| Neri et al. 2018 [26] | 0 | 0 | 0 | 1 | 0 | 1 | 0 | 1 | 0 | **3 (Poor)** |  |
| O'Neil-Callahan et al. 2013 [23] | 1 | 1 | 1 | 1 | 1 | 1 | 0 | 1 | 0 | **7 (Poor)** |  |
| Peipert et al. 2011 [24] | 1 | 1 | 1 | 1 | 1 | 1 | 0 | 1 | 0 | **7 (Good)** |  |
| Phillips et al. 2017 [44] | 1 | 1 | 1 | 0 | 1 | 1 | 1 | 1 | 0 | **7 (Good)** |  |
| Piva et al. 2019 [51] | 0 | 1 | 1 | 0 | 1 | 1 | 0 | 1 | 1 | **6 (Fair)** |  |
| Sanders et al. 2018 [53] | 1 | 0 | 1 | 1 | 0 | 1 | 1 | 1 | 0 | **6 (Good)** |  |
| Shimoni et al. 2019 [25] | 1 | 1 | 1 | 1 | 1 | 1 | 0 | 1 | 0 | **7 (Good)** |  |
| Short et al. 2014 [56] | 1 | 1 | 1 | 1 | 1 | 1 | 1 | 1 | 1 | **8 (Good)** |  |
| Vaitsiakhovich et al. 2018 [27] | 1 | 0 | 0 | 0 | 1 | 0 | 0 | 0 | 0 | **2 (Poor)** |  |
| Weisberg et al. 2014 [57] | 1 | 0 | 1 | 1 | 1 | 1 | 0 | 1 | 0 | **7 (Good)** |  |
| Wiebe and Trussell 2016 [38] | 1 | 0 | 1 | 1 | 1 | 1 | 0 | 1 | 0 | **6 (Poor)** |  |
| Wildemeersch et al. 2014 [47] | 1 | 1 | 1 | 1 | 1 | 1 | 0 | 1 | 0 | **7 (Poor)** |  |

^a^Thresholds for converting the Newcastle–Ottawa scales to Agency for Healthcare Research and Quality standards (good, fair, and poor):

**Good quality:** 3 or 4 stars in selection domain AND 1 or 2 stars in comparability domain AND 2 or 3 stars in outcome/exposure domain;

**Fair quality:** 2 stars in selection domain AND 1 or 2 stars in comparability domain AND 2 or 3 stars in outcome/exposure domain;

**Poor quality:** 0 or 1 star in selection domain OR 0 stars in comparability domain OR 0 or 1 stars in outcome/exposure domain.

Table C. Detailed Newcastle–Ottawa Scale of case-control/RCT studies included

| **Study** | **Selection** | | | | | **Comparability** | **Exposure** | | |  |
| --- | --- | --- | --- | --- | --- | --- | --- | --- | --- | --- |
|  | **Case definition** | **Representative- ness** | **Selection of Controls** | **Definition of Controls** | **Design or analysis** | | **Ascertain-ment of exposure** | **Ascertain-ment for cases and controls** | **Non-Response rate** | **Total score**  **(quality)^a^** |
| Akintomide et al. 2019 [42] | 0 | 1 | 0 | 1 | 1 | | 0 | 1 | 0 | **4 (Poor)** |
| Apter et al. 2016 [54] | 1 | 1 | 0 | 1 | 0 | | 0 | 1 | 0 | **4 (Poor)** |
| Eisenberg et al. 2015 [12] | 1 | 1 | 0 | 1 | 1 | | 0 | 1 | 0 | **5 (Poor)** |
| Gemzell et al. 2015 [17] | 1 | 1 | 0 | 1 | 1 | | 1 | 1 | 1 | **7 (Good)** |
| Korjamo et al. 2017 [20] | 1 | 1 | 0 | 0 | 0 | | 1 | 1 | 0 | **4 (Poor)** |
| Korjamo et al. 2017 [20] | 1 | 1 | 0 | 0 | 0 | | 1 | 1 | 1 | **5 (Poor)** |
| Modesto et al. 2014 [55] | 1 | 1 | 0 | 0 | 2 | | 0 | 0 | 1 | **5 (Poor)** |
| Maguire et al. 2015 [46] | 1 | 1 | 0 | 1 | 2 | | 1 | 1 | 1 | **8 (Good)** |
| Nelson et al. 2013 [18] | 1 | 1 | 0 | 1 | 2 | | 1 | 1 | 1 | **8 (Good)** |
| Reeves et al. 2017 [41] | 1 | 1 | 0 | 1 | 2 | | 1 | 1 | 0 | **7 (Good)** |
| Sanders et al. 2018 [53] | 1 | 1 | 0 | 0 | 2 | | 0 | 1 | 0 | **5 (Poor)** |
| Schreiber et al. 2018 [13] | 1 | 0 | 1 | 1 | 1 | | 0 | 1 | 1 | **6 (Good)** |
| Shimoni et al. 2011 [40] | 1 | 1 | 1 | 0 | 1 | | 0 | 1 | 0 | **5 (Poor)** |
| Short et al. 2012 [58] | 1 | 1 | 1 | 1 | 2 | | 0 | 0 | 0 | **6 (Poor)** |
| Teal et al. 2019 [14] | 1 | 1 | 0 | 1 | 1 | | 1 | 1 | 0 | **6 (Good)** |
| Whitaker et al. 2014 [30] | 1 | 1 | 0 | 1 | 2 | | 1 | 1 | 0 | **7 (Good)** |
| Yaron et al. 2019 [33] | 1 | 0 | 1 | 1 | 1 | | 1 | 0 | 1 | **6 (Good)** |

^a^Thresholds for converting the Newcastle–Ottawa scales to Agency for Healthcare Research and Quality standards (good, fair, and poor):

**Good quality:** 3 or 4 stars in selection domain AND 1 or 2 stars in comparability domain AND 2 or 3 stars in outcome/exposure domain;

**Fair quality:** 2 stars in selection domain AND 1 or 2 stars in comparability domain AND 2 or 3 stars in outcome/exposure domain;

**Poor quality:** 0 or 1 star in selection domain OR 0 stars in comparability domain OR 0 or 1 stars in outcome/exposure domain.

Table D. Detailed Newcastle–Ottawa Scale of cross-sectional studies included

| **Study** | **Selection** | | | | **Comparability** | **Outcome** | |  |
| --- | --- | --- | --- | --- | --- | --- | --- | --- |
|  | **Representative of the sample** | **Sample size** | **Non-respondents** | **Ascertainment of the exposure (risk factor)** | **Confounding factors controlled** | **Assessment of outcome** | **Statistical test** | **Total score**  **(quality)^a^** |
| Scavuzzi et al. 2016 [37] | 1 | 1 | 0 | 1 | 2 | 0 | 1 | 6 (satisfactory) |
| Stoegerer-Hecher et al. 2012 [32] | 1 | 1 | 0 | 1 | 2 | 1 | 0 | 6 (satisfactory) |

^a^Thresholds for converting the Newcastle–Ottawa scales to Agency for Healthcare Research and Quality standards:

**Very Good Study**: 9–10 points
**Good Study**: 7–8 points

**Satisfactory Study**: 5–6 points

**Unsatisfactory Study**: 0–4 points
